# Histogram-derived modified thresholds for coronary artery calcium scoring with lower tube voltage

**DOI:** 10.1038/s41598-021-96695-9

**Published:** 2021-08-31

**Authors:** Sungwon Kim, Chan Joo Lee, Kyunghwa Han, Kye Ho Lee, Hye-Jeong Lee, Sungha Park

**Affiliations:** 1 Department of Radiology, Research Institute of Radiological Science, Severance Hospital, Yonsei University College of Medicine, 50-1 Yonsei-ro, Seodaemun-gu, Seoul, 03722 Republic of Korea; 2 Division of Cardiology, Severance Cardiovascular Hospital and Integrative Research Center for Cerebrovascular and Cardiovascular Diseases, Yonsei University College of Medicine, 50-1 Yonsei-ro, Seodaemun-gu, Seoul, 03722 Republic of Korea

**Keywords:** Cardiology, Medical research

## Abstract

We aimed to determine the proper modified thresholds for detecting and weighting CAC scores at 100 kV through histogram matching in comparison with 120 kV as a standard reference. From the training set (680 participants), modified thresholds at 100 kV were obtained through histogram matching of calcium pixels to 120 kV. From the validation set (213 participants), a standard CAC score at 120 kV, and modified CAC score at 100 kV using modified thresholds were compare through the paired *t* test and the Bland–Altman plot. Agreement for risk categories (no, minimal, mild, moderate, and severe) was evaluated using kappa statistics. Radiation doses were also compared. For the validation set, there was no significant difference between standard (median, 18.7; IQR, 0.0–207.0) and modified (median, 17.3; IQR, 0.0–220.9) CAC scores (*P* = 0.689). A small bias was achieved (0.74) with 95% limits of agreement from − 52.35 to 53.83. Agreements for risk categories were excellent (κ = 0.994). The mean dose-length-product of 100-kV scanning (30.1 ± 0.8 mGy * cm) was significantly decreased compared to 120-kV scanning (42.9 ± 0.6 mGy * cm) (*P* < 0.001). Histogram-derived modified thresholds at 100 kV can enable accurate CAC scoring while reducing radiation exposure.

## Introduction

Non-contrast cardiac computed tomography (CT) is used to determine the presence and extent of calcified atherosclerotic burdens in the coronary arteries and this burden is quantified as the coronary artery calcium (CAC) score^1^. The CAC score is a well-established reliable and reproducible predictor of coronary artery disease, and its addition to traditional risk assessment models has provided incremental information to predict future outcomes in certain populations^2^. Hence, recent guidelines recommend CAC scoring to determine when to initiate statin therapy in intermediate-risk patients^3^.

Formal CAC scoring was introduced in 1990 by Agatston et al.^4^. The Agatston score is not only easy to obtain with acceptable inter-scan and inter-observer reproducibility, cumulative clinical evidence supports its use for risk stratification in primary prevention settings^2^. Hence, it remains the gold standard and the most commonly used CAC score in clinical practice even though the Agatston scores tend to show a large degree of arbitrariness^5^. However, the potential benefits of CAC scoring need to be weighed against its potential risks, along with reflections on exposure to ionizing radiation. The current trend is to gradually lower radiation exposure during CT scans following the ‘as low as reasonably achievable (ALARA)’ principle because of public concerns due to the theoretical association between cancer and ionizing radiation^6^. In contrast to coronary CT angiography which now incorporates advanced imaging acquisition and reconstruction techniques to reduce radiation exposure, the CAC scanning protocol remains largely unchanged from the initial technique proposed in 1990, especially for tube voltage settings. Lowering tube voltage remains a challenge for CAC scoring because the CT attenuation of calcium is closely related to photon energy; thus the thresholds established by Agatston are not applicable to other tube voltages^7^. Nevertheless, prior researchers have studied the feasibility of lower tube voltages in CAC scoring since a marked reduction of radiation dose can be achieved. However, there have been conflicting results as prior studies include only small populations and are based on different assumptions on coronary calcifications. Therefore, we aimed to determine modified thresholds appropriate for detecting and weighting CAC scores at 100 kV through an intuitive and eidetic method using histograms with a large-scale population and temporal independent validation.

## Methods

### Study population

The study protocol adhered to the principles of the Declaration of Helsinki, and the institutional review board of Severance Hospital, Yonsei university-affiliated tertiary referral hospital, approved this prospective study (IRB 4-2013-0581). All study participants gave informed consent. We prospectively included 902 participants from the Cardiovascular and Metabolic Disease Etiology Research Center-High Risk Cohort (CMERC-HI; clinicaltrials.gov: NCT02003781) from January 2017 to July 2018^8^. These participants were divided two data sets with consecutive cohorts based on a specific point in time for temporal validation^9^. The first (training) set consisted of consecutive participants from January 2017 to February 2018, and the second (temporal independent validation) consisted of consecutive participants from March 2018 to July 2018.

### CAC scanning

CAC scans were performed with the latest 256-slice CT scanner (Revolution, GE Healthcare) for all study participants, and the scan consisted of standard scanning at 120 kV and additional scanning at 100 kV. Tube currents were set to 200 mA for both scans. All scans were done with prospective ECG-gated acquisitions at mid-diastole (70% of the R-R interval). Other scanning parameters were a 512 × 512 pixel matrix, 256 × 0.625 mm slice collimation, and 0.28 s rotation time. Scan range and field of view were adjusted according to heart size. After scanning, axial images were reconstructed with a 2.5 mm slice thickness and 2.5 mm increment interval through a medium-smooth convolution kernel using filtered back projection. Volumetric CT dose index (CTDI) and dose-length-product (DLP) were recorded. Effective radiation doses were estimated using a conversion factor for cardiac CT (0.026 mSv/mGy * cm)^10^.

### Image analysis

All CT images were transferred to a commercially available workstation (Aquarius iNtuition V4.4.6; TeraRecon). An observer (H-J. L., 12 years of experience in cardiac imaging) measured the signal-to-noise ratio (SNR) from CT images. The proximal ascending aorta was evaluated at the level of the main pulmonary artery bifurcation using an approximately 200 mm^2^ circular region of interest to measure mean attenuation and standard deviation in Hounsfield units (HU). Afterward, all cardiac CT images were transformed to the NRRD (Nearly Raw Raster Data) files from the DICOM (Digital Imaging and Communications in Medicine) imaging data set using the Insight Segmentation and Registration Toolkit, or ITK, package (V5.2.0) (https://www.itk.org)^11^. Our personal computer-based in-house software program (V1.1) in MATLAB (Matlab R2018a; Mathworks) was used for CAC scoring with the NRRD files. Contiguous voxels ≥ 1 mm^2^ in areas with CT attenuation ≥ a threshold of 130 HU were automatically colored as calcifications by the in-house software for both 100 kV and 120 kV images (Supplementary Fig. S1). As the observer selected colored lesions along the coronary vessels, information on the total lesion area with volume, and density in pixels were collected as the CAC score was calculated. To validate our in-house software, the observer measured the CAC score for a selected population of the training set using a commercially available workstation (Aquarius iNtuition V4.4.6; TeraRecon) as the reference standard, and inter-test agreements were assessed. For inter-observer agreement, an additional observer (K. H. L., 7 years of experience in cardiac imaging) evaluated CT images using the in-house software for the same selected population in both 100 kV and 120 kV images. CAC scoring was based on the Agatston score as described previously^4^.

### Modified thresholds for CAC scoring at 100 kV

**Figure 1:**
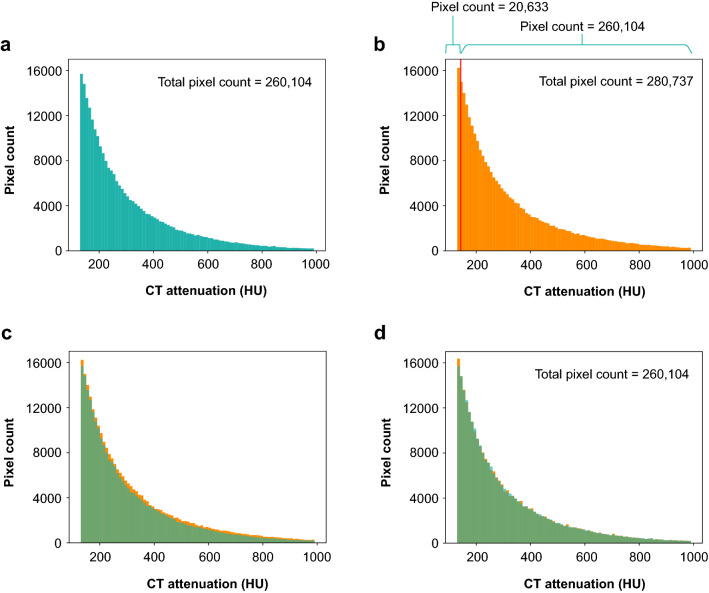
Histogram matching using the cumulative distribution function to obtain modified thresholds for 100 kV. The 120 kV histogram showed CT numbers of 130 HU through 1887 HU for 260,104 pixels from coronary calcium (**a**). The 100 kV histogram demonstrated 280,737 pixels with CT numbers from 130 HU to 2059 HU (**b**). The overlapping two histograms (**a**,**b**) showed more pixels from 100 kV images (**c**). After excluding 20,633 pixels (280,737 – 260,104 = 20,633) from the 130 HU of the 100 kV histogram, the two histograms were almost identical (**d**). Afterwards, we found matching points in the 100 kV histogram.

All processing steps were conducted in Python (version 3.6.6; Python Software Foundation), and are described in Fig. 1. From the training set, all pixels more than 130 HU of coronary calcium on 120 kV and 100 kV images were selected, and arranged according to HU. As mentioned earlier, the attenuation of calcium is higher at 100 kV, so there were more pixels on 100 kV images. Pixels near 130 HU on 100 kV images would not be recognized at 120 kV. Therefore, we selected pixels from 100 kV images in descending order from the largest HU when counting the total number of calcium pixels between 120 and 100 kV. Afterwards, we plotted histograms for the selected pixels of the 100 kV images and performed histogram matching using the cumulative distribution function with the 120 kV histogram^11^. Points in the 100 kV histogram that corresponded to 130, 200, 300, and 400 HU in the 120 kV histogram were identified for the modified thresholds with 100 kV. The standard CAC score was calculated from 120 kV images with the original thresholds of 130, 200, 300, and 400 HU. The modified CAC score was calculated from 100 kV images with the newly obtained modified thresholds. In addition, based on the standard and modified CAC scores respectively, each participant was classified into the following risk categories: no calcium (CAC score = 0), minimal (≤ 10), mild (> 10, ≤ 100), moderate (> 100, ≤ 400), severe (> 400)^12^.

### Statistical analysis

Statistical analyses were performed using commercially available statistical software (SPSS, version 25.0; IBM SPSS Statistics). CAC scores were presented as median with corresponding interquartile range (IQR). Other continuous variables were described as means with standard deviation after confirming normality through the Shapiro–Wilk test. Categorical variables were expressed as the participant numbers. A subgroup population was selected from the training set by random number generation to validate the in-house software and evaluate inter-observer agreements. Inter-test agreements for software validation and inter-observer agreements were analyzed with the intraclass correlation coefficient (ICC). Baseline characteristics were compared between the training set and validation set with the Student *t* test and the Pearson’s chi-square test. Between 100 and 120 kV, CT characteristics were compared with the paired *t* test and median values of standard and modified CAC scores were compared with Wilcoxon’s signed rank test. Additionally, Bland–Altman plot were used to calculate agreements between the both CAC scores. Agreements for risk categories between the two scores was evaluated using kappa statistics and contingency tables. A *P* value < 0.05 was interpreted as being statistically significant.

## Results

### Baseline characteristics

Of 902 participants, 9 were excluded because technical problems did not allow their CT images to be processed for this study. Thus, 893 participants were included and grouped into two data sets. The first data set (training) consisted of 680 consecutive participants (363 men and 317 women; mean age, 58.2 ± 11.3 years; range, 20–80 years). The second data set (temporal validation) consisted of 213 consecutive participants (102 men and 111 women; mean age, 58.1 ± 11.0 years; range, 26–80 years). Baseline clinical characteristics of the study population are summarized in Table 1.

**Table 1:** Clinical characteristics of participants in the training and validation sets.

Variables	Total (n = 893)	Training (n = 680)	Validation (n = 213)	*P* value
Male	465 (52.1%)	363 (53.4%)	102 (47.9%)	0.161
Age (years)	58.1 ± 11.5	58.2 ± 11.3	58.1 ± 11.0	0.985
BMI (kg/m^2^)	26.0 ± 3.6	26.0 ± 3.5	25.9 ± 3.9	0.977
SBP (mmHg)	126.9 ± 16.4	127.2 ± 17.0	125.9 ± 14.4	0.313
DBP (mmHg)	76.5 ± 10.3	76.9 ± 10.3	75.3 ± 10.2	0.054
**Medical history**				
Smoking	126 (14.1%)	95 (14.0%)	31 (14.6%)	0.831
Hypertension	718 (80.4%)	565 (83.1%)	153 (71.8%)	< 0.001
Diabetes	521 (58.3%)	366 (53.8%)	155 (72.8%)	< 0.001
Dyslipidemia	639 (71.6%)	462 (67.9%)	177 (83.1%)	< 0.001
**Laboratory findings**				
Fasting glucose (mg/dL)	120.4 ± 36.8	119.5 ± 34.8	123.2 ± 42.7	0.196
Total cholesterol (mg/dL)	173.1 ± 43.3	175.1 ± 45.2	166.9 ± 35.8	0.016
BUN (mg/dL)	21.2 ± 13.8	21.8 ± 14.6	19.1 ± 10.6	0.011
Serum creatinine (mg/dL)	1.4 ± 1.7	1.5 ± 1.9	1.1 ± 1.1	0.014

Data are absolute participant numbers and percentages in brackets or means ± standard deviations.

*BMI* body mass index, *SBP* systolic blood pressure, *DBP* diastolic blood pressure, *BUN* blood urea nitrogen.

Baseline CT characteristics are described in Table 2. For the training set, DLP was 30.1 ± 0.8 mGy * cm in 100 kV and 42.9 ± 0.6 mGy * cm in 120 kV. Corresponding effective radiation dose was 0.42 ± 0.01 and 0.60 ± 0.01 mSv, respectively. The mean SNRs of 100 kV and 120 kV were 1.55 ± 0.19 and 1.63 ± 0.23, respectively. For the validation set, DLP was 30.1 ± 0.8 mGy * cm in 100 kV and 42.9 ± 0.6 mGy * cm for 120 kV. Corresponding effective radiation dose was 0.42 ± 0.01 and 0.60 ± 0.01 mSv, respectively. The mean SNRs of 100 kV and 120 kV were 1.57 ± 0.20 and 1.66 ± 0.26, respectively. For both training and validation sets, even though SNR significantly decreased in 100 kV (*P* < 0.001 for both sets), the radiation dose showed significant reduction compared to 120 kV (*P* < 0.001 for both sets). Baseline CT characteristics did not differ between the training and validation sets.

**Table 2:** CT characteristics of participants in the training and validation sets.

	Total (n = 893)	Training set (n = 680)	Validation set (n = 213)	*P* value^a^
Heart rates (bpm)	66.4 ± 11.5	66.0 ± 11.4	67.7 ± 11.7	0.070
**CTDI (mGy)**				
100 kV	1.9 ± 0.1	1.9 ± 0.1	1.9 ± 0.1	0.234
120 kV	2.8 ± 0.0	2.8 ± 0.1	2.8 ± 0.0	0.622
*P* value^b^	< 0.001	< 0.001	< 0.001	
DLP (mGy * cm)				
100 kV	30.1 ± 0.8	30.1 ± 0.8	30.1 ± 0.8	0.234
120 kV	42.9 ± 0.6	42.9 ± 0.6	42.9 ± 0.6	0.622
*P* value^b^	< 0.001	< 0.001	< 0.001	
**Signal (HU)**				
100 kV	43.4 ± 4.3	43.3 ± 4.2	43.9 ± 4.3	0.073
120 kV	42.1 ± 4.8	42.0 ± 4.8	42.5 ± 4.7	0.114
*P* value^b^	< 0.001	< 0.001	0.001	
**Noise (HU)**				
100 kV	28.2 ± 2.6	28.2 ± 2.7	28.1 ± 2.5	0.809
120 kV	25.9 ± 2.9	25.9 ± 2.9	26.0 ± 2.8	0.955
*P* value^b^	< 0.001	< 0.001	< 0.001	
**Signal-to-noise ratio**				
100 kV	1.55 ± 0.20	1.55 ± 0.19	1.57 ± 0.20	0.111
120 kV	1.64 ± 0.24	1.63 ± 0.23	1.66 ± 0.26	0.154
*P* value^b^	< 0.001	< 0.001	< 0.001	

Data are means ± standard deviations.

*CTDI* volumetric CT dose index, *DLP* dose-length-product.

^a^Between the training set and validation set.

^b^Between the 100-kV and 120-kV images.

### Modified CAC scoring at 100 kV

From the training set, the median standard CAC score at 120 kV was 17.6 (IQR, 0.0–164.5), and 250 (36.8%, 250/680) participants had scores of zero. The 120 kV histogram showed a wide range of CT numbers from 130 HU to 1887 HU for 260,104 pixels of coronary calcium. A total of 280,737 pixels from coronary calcium at 100 kV demonstrated CT numbers from 130 HU to 2059 HU (Fig. 1). Through histogram matching after arranging pixels, we obtained modified thresholds of 143 HU for calcium detection instead of 130 HU, and additionally 220 HU, 329 HU, and 439 HU for weighting scores of 2, 3, and 4 at 100 kV, respectively.

**Figure 2:**
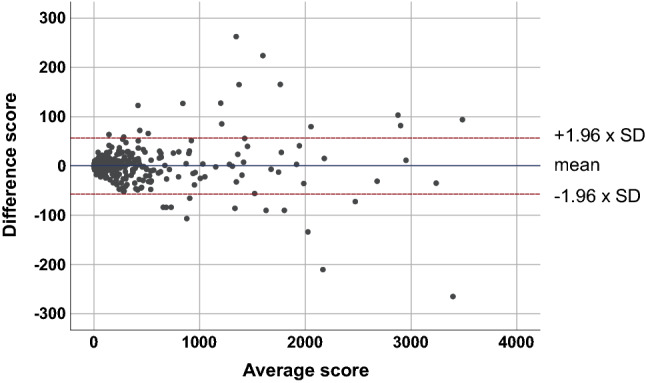
Bland–Altman plot between the standard and modified CAC scores from the training set (n = 680).

The median modified CAC score with the modified threshold at 100 kV was 18.6 (IQR, 0.0–159.9). There was no significant difference between the median values of the standard and modified CAC scores (*P* = 0.696). A small bias was calculated (0.65), with 95% limits of agreement of − 57.04 and 58.34 through the Bland–Altman plot (Fig. 2). Agreements between the standard and modified CAC scores for risk categories are shown in Table 3. For the training set, 29 (4.2%, 29/680) participants showed changes in risk categories with the modified CAC score, and excellent agreement (κ = 0.943) between the standard and modified CAC scores.

**Table 3:** Agreements for risk categories between the standard and modified CAC scores from the training set (κ = 0.943).

Modified CAC score	Standard CAC score					
	No	Minimal	Mild	Moderate	Severe	Total
No	249	5	0	0	0	254
Minimal	1	54	3	0	0	58
Mild	0	7	145	5	0	157
Moderate	0	0	3	113	3	119
Severe	0	0	0	2	90	92
Total	250	66	151	120	93	680

Data are absolute participant numbers.

*CAC* coronary artery calcium.

### Inter-test and inter-observer agreements

The subpopulation from the training set selected for the inter-test agreement and inter-observer agreements consisted of 70 participants (10.3%, 70/680) (37 men and 33 women; mean age, 56.5 ± 12.6 years; range, 20–78 years). Inter-test agreement was excellent (ICC = 1.000) for the standard CAC score between our in-house software (median, 14.9; IQR, 0.0–69.9) and the commercially available workstation (median, 15.2; IQR, 0.0–70.3). The median standard CAC score of the additional observer using our software for the same subpopulation was 14.9 (IQR, 0.0–69.9), and inter-observer agreement was excellent (ICC = 1.000). The median values of the modified CAC score for the two observers were 14.9 (IQR, 0.0–70.0) and 15.1 (IQR, 0.0–70.0), respectively, and inter-observer agreement was excellent (ICC = 1.000).

### Temporal independent validation

**Figure 3:**
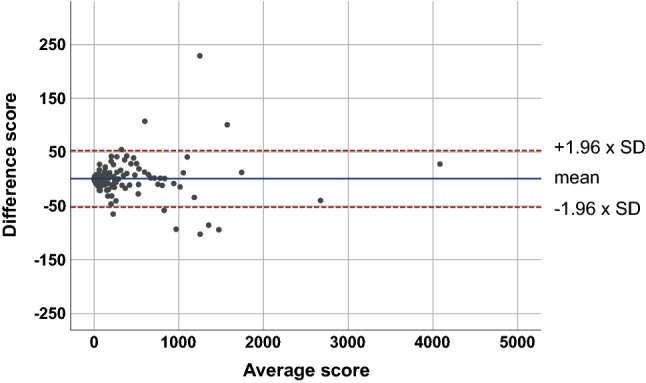
Bland–Altman plot between the standard and modified CAC scores from the validation set (n = 213).

In the validation set, 83 (39.0%, 83/213) participants showed no coronary calcifications. The median standard and modified CAC scores were 18.7 (IQR, 0.0–207.0) and 17.3 (IQR, 0.0–220.9), respectively, and there was no significant difference between the CAC scores (*P* = 0.689). In addition, the values of the validation set did not significantly differ from those of the training set (*P* = 0.415 and 0.416, respectively). A small bias was achieved (0.74), with 95% limits of agreement from − 52.35 to 53.83 (Fig. 3). Sub-analyses were additionally performed, and the results are described in Supplementary material. Agreements for risk categories between the standard and modified CAC scores are demonstrated in Table 4. In the validation set, only 2 (0.9%, 2/213) participants had their risk category change with the modified CAC score. Agreement was excellent (κ = 0.994) between the standard and modified CAC scores for risk categories.

**Table 4:** Agreement for risk categories between the standard and modified CAC scores from the validation set (κ = 0.994).

Modified CAC score	Standard CAC score					
	No	Minimal	Mild	Moderate	Severe	Total
No	82	0	0	0	0	82
Minimal	1	14	1	0	0	16
Mild	0	0	36	0	0	36
Moderate	0	0	0	46	0	46
Severe	0	0	0	0	33	33
Total	83	14	37	46	33	213

Data are absolute participant numbers.

*CAC* coronary artery calcium.

**Figure 4:**
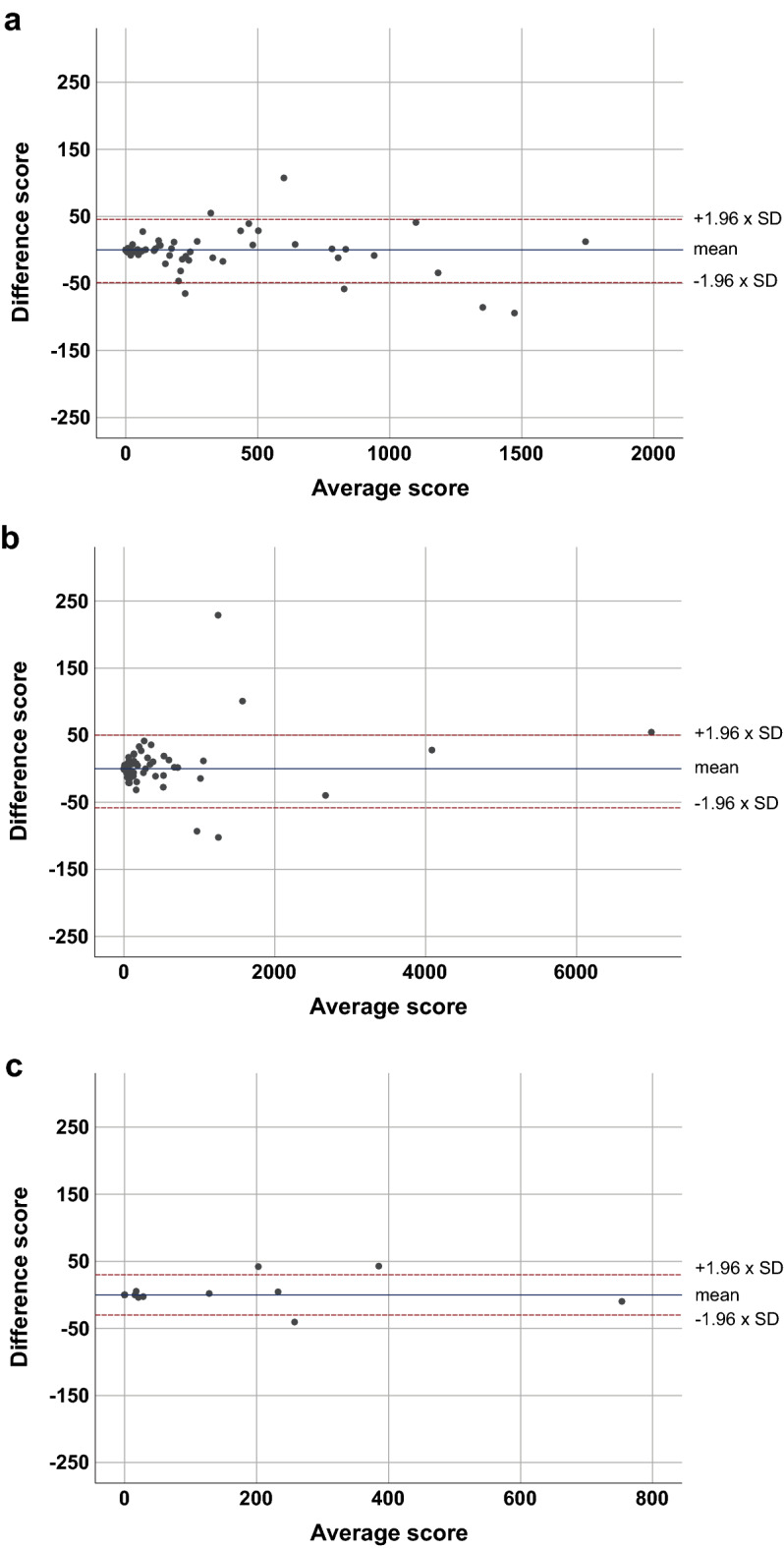
Bland–Altman plot between the standard and modified CAC scores from sub-analysis according to BMI. (**a**) BMI < 25 kg/m^2^, (**b**) 25 ≤ BMI < 30 kg/m^2^, and (**c**) BMI ≥ 30 kg/m^2^.

A sub-analysis was performed according to the BMI (Fig. 4). In a sub-analysis of 89 participants with BMI < 25 kg/m^2^ (median, 23.1; IQR, 21.6–24.2) (42.8%, 89/213), there was no significant difference between the standard and modified CAC scores (median, 29.4; IQR, 0.0–222.3, and median, 22.6; IQR, 0.0–245.5, respectively) (*P* = 0.439). Thirty-seven participants (41.6%, 37/89) showed no coronary calcifications. A bias of − 1.62 with 95% limits of agreement from 45.46 to − 48.72 was observed. For the 103 participants with BMI ≥ 25 and < 30 kg/m^2^ (median, 26.9; IQR, 26.0–28.2) (48.4%, 103/213), the median standard and modified CAC scores were 25.9 (IQR, 0.0–175.2) and 22.9 (IQR, 0.0–178.5), respectively, without significant difference (*P* = 0.462). No coronary calcification was observed in 38 participants (36.9%, 38/103). The bias was 2.55 with 95% limits of agreement from 63.47 to − 58.36. In the 21 participants with BMI ≥ 30 kg/m^2^ (median, 32.8; IQR, 31.4–37.2) (9.9%, 21/213), no significant difference was observed between the median standard and modified CAC scores (median, 12.5; IQR, 0.0–129.3, and median, 13.7; IQR, 0.0–127.2, respectively) (*P* = 0.646). Eight participants (38.1%, 8/21) showed no coronary calcifications. A bias of 1.91 was obtained with 95% limits of agreement from 33.95 to − 30.13.

## Discussion

Through our study, we were able to attain modified thresholds for CAC scoring with 100 kV from histograms using the cumulative distribution function, and CAC scoring was accurate with good agreement of a small bias and acceptable 95% limits of agreement compared to standard CAC scoring with 120 kV. In addition, we obtained excellent agreement for risk categories between the standard and modified CAC scores. Further, these findings were confirmed through temporal independent validation with similar results. As expected, the SNR significantly decreased at 100 kV compared to 120 kV, but the reduction rate was about 5.5%. Foremost, the mean radiation dose of 100 kV scanning was further decreased with a reduction rate of 30.0%.

Although a few studies have been conducted to assess the feasibility of lower tube voltages in CAC scanning, the results of these studies have not been applied in clinical practice, mostly due to the small size of their study populations and insufficient validation. Hence, there have been conflicting results on the application of lower tube voltages to CAC scanning despite the great reduction of radiation dose being a common compelling finding. In prior studies, the detection threshold for coronary calcifications was adjusted to 147 HU instead of 130 HU at 100 kV without adjusting weighting thresholds. One of these past studies showed equivalent results compared with a standard protocol at 120 kV while another study reported a systematic bias toward overestimation of the Agatston score^7,13^. The threshold of 147 HU was determined from phantom data by simply calculating the ratio of plaque attenuation at 100 kV to 120 kV. Similarly, a few studies with phantoms composed of several calcium hydroxyapatite pieces were done to evaluate the feasibility of lower tube voltages^14–18^. However, we have to interpret these results with caution, because the size and degree of coronary calcium varies in vivo, and it is thought difficult for studies to reflect this broad diversity. In a more recent study, researchers calculated mathematically derived novel thresholds for CAC scores at lower tube voltages by assuming a linear relationship between attenuation coefficients and photon energy in a relatively low energy spectrum and calculated the ratio between the attenuation coefficients at standard 120 kV photon energy versus lower kV^19^. In contrast, we used a more intuitive and eidetic method to obtain modified thresholds. We split actual CT attenuations from coronary calcifications in vivo into lots of pixels, displayed the values, and matched points of CT attenuations with the highest probability between 100 and 120 kV for the detection and weighting thresholds. We obtained modified thresholds of 143 HU, 220 HU, 320 HU, and 439 HU that were comparable to the mathematically derived thresholds of 145 HU, 223 HU, 335 HU, and 447 HU. We supposed that our results were slightly lower than those of the prior study because the attenuation coefficient and photon energy showed a somewhat downward exponential relationship rather than a true linear one. In our results, we obtained good agreement between the standard and modified CAC scores with a small bias and acceptable 95% limits of agreement compared to prior studies at 100 kV^7,13,20,21^. Moreover, we showed better agreement for risk categories between 100 and 120 kV with modified thresholds as we obtained higher kappa values^7^ and lower percentages of participants that had their risk category change^13,20,21^ compared to prior studies, although a direct comparison with previous studies is currently difficult to perform. Of course, a few studies using tin-filtered 100 kV showed good results in comparison with 120 kV for CAC scoring^20,21^, but there are limitations to the general use of this technique because it is a scanner-specific tool.

Although we regarded 100 kV as low tube voltage, a few prior studies have evaluated the feasibility of the lower 70 kV or 80 kV for CAC scoring^19,22^. However, although these protocols are useful for small-sized patients, they do not seem applicable for large-sized patients as increased image noise would affect the CAC score. On the other hand, body size had little effect on the CAC score in the present study at 100 kV as similar results of bias and 95% limits of agreement were found among subgroups classified according to the BMI. Still, more studies are needed to further lower tube voltage or select BMI-adapted tube voltage appropriate for CAC scoring that can also achieve great reduction of radiation exposure.

Our study has several limitations. First, even though our sample size was relatively large, a large portion of the participants did not have coronary calcium. Because consecutive participants with no prior history of coronary artery disease were prospectively enrolled, it was not possible to limit the number of participants with no coronary calcium. However, we obtained similar results even after excluding participants with no coronary calcium from the validation set. Second, the study population consisted of a single ethnic Korean population with relatively small body size, which inherently limited the generalizability of the study findings. In addition, we studied the findings on a single CT scanner. Future studies are needed to investigate the feasibility of applying the modified thresholds to different clinical settings that include patients of diverse body sizes. Third, misregistration errors could exist between the 100 kV and 120 kV CT images despite separating CT attenuation from coronary calcifications into pixels. The dual-energy technique might help solve misregistration in future studies.

In conclusion, the present study suggests that histogram-derived modified thresholds at 100 kV could allow the accurate calculation of CAC scores while still managing to reduce radiation exposure. Our findings are further strengthened by the relative large scale of our population and temporal independent validation. Further studies using the modified thresholds on different scanning systems might be needed before CAC scoring can become an actual clinical utility.

## Supplementary Information

Supplementary Information.

Supplementary Figure S1.

Supplementary Figure S2.

## Data Availability

The datasets used and/or analyzed during the current study are available from the corresponding author on reasonable request.
