# Supplementary material for: Histogram-derived modified thresholds for coronary artery calcium scoring with lower tube voltage
Source: Sci Rep. 2021 Aug 31;11:17450. doi: 10.1038/s41598-021-96695-9 (PMC8408203; doi:10.1038/s41598-021-96695-9)
Supplement: Supplementary file 1 — Supplementary Information. [file 41598_2021_96695_MOESM1_ESM.docx]

**Histogram-derived modified thresholds for coronary artery calcium scoring with lower tube voltage**

Sungwon Kim, MD, PhD^1,3^, Chan Joo Lee, MD, PhD^2,3^, Kyunghwa Han, PhD^1^, Kye Ho Lee, MD^1^, Hye-Jeong Lee, MD, PhD^1*^, Sungha Park, MD, PhD^2*^

**Supplementary material**

**Results**

A sub-analysis that excluded participants with a standard CAC score of zero (130 participants; 61.0%, 130/213) was performed. The median standard CAC score was 142.3 (IQR, 35.3– 411.3) and modified CAC score was 145.2 (IQR, 38.2–410.7). There was no significant difference in the median values between two scores (*P* = 0.839). A bias of 1.23 with 95% limits of agreement from -66.81 to 69.27 was observed (Supplementary Fig. S2).

**Figures and Figure legends**

**Supplementary Figure S1.** A representative case of CAC scoring in a 75-year-old female patient with a BMI of 30.4 kg/m^2^. The standard CAC score of this patient was 386.6 (a). After the modified thresholds were applied, the modified CAC score with 100 kV was 363.7 (b).

**Supplementary Figure S2.** Bland-Altman plot between the standard and modified CAC scores from participants with excluding those of zero coronary calcifications.
