# Supplementary figures and images for: Histogram-derived modified thresholds for coronary artery calcium scoring with lower tube voltage
Source: Sci Rep. 2021 Aug 31;11:17450. doi: 10.1038/s41598-021-96695-9 (PMC8408203; doi:10.1038/s41598-021-96695-9)

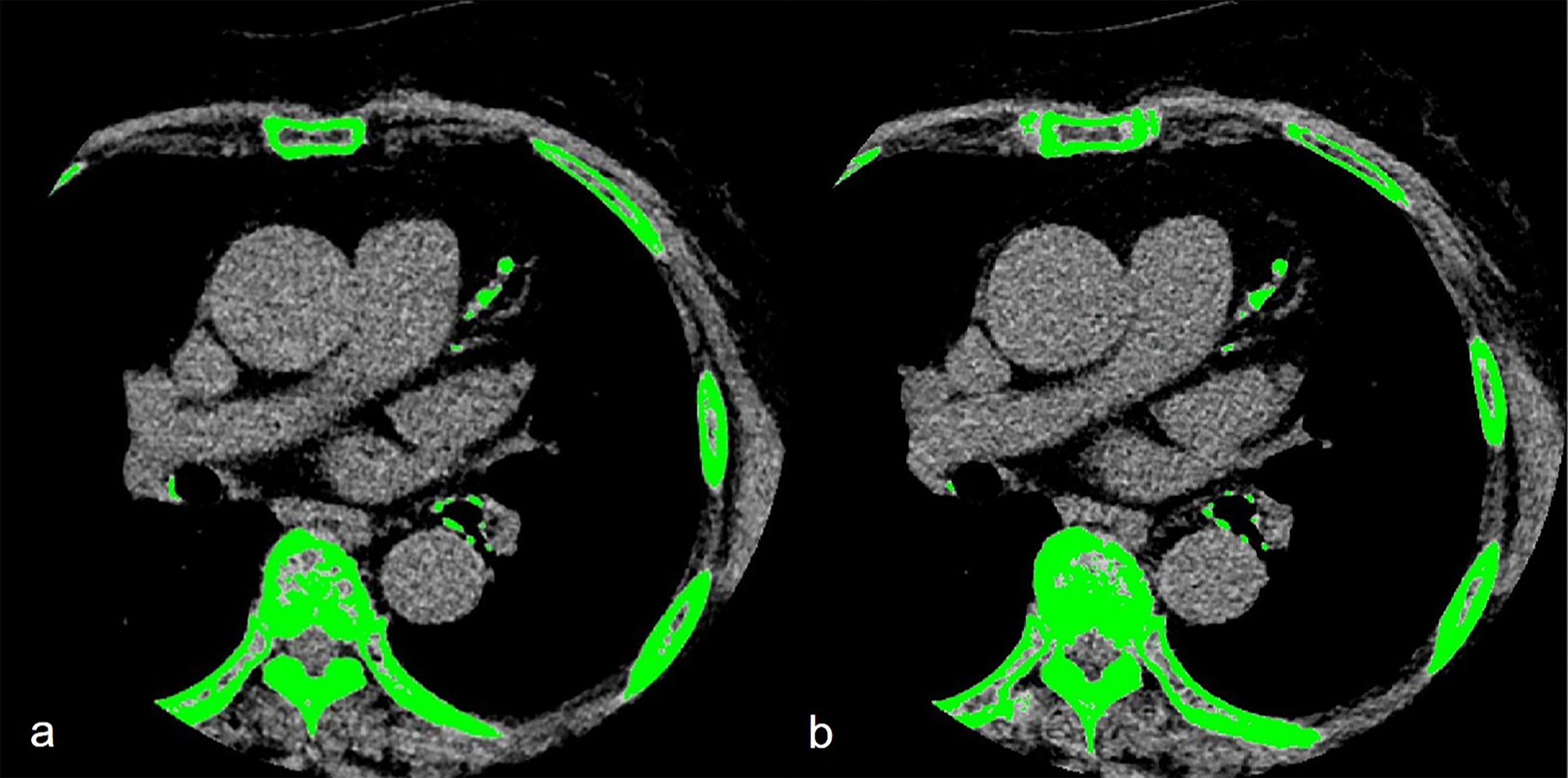

Supplement: Supplementary file 2 — Supplementary Figure S1. [file 41598_2021_96695_MOESM2_ESM.tif]
